# Acute Rhabdomyolysis in a Child with Multiple Suspicious Gene Variants

**DOI:** 10.1155/2022/2099827

**Published:** 2022-09-24

**Authors:** Aiko Murakami, Rhiana L. Lau, Robert Wallerstein, Tamara Zagustin, Garett Kuwada, Prashant J. Purohit

**Affiliations:** ^1^Department of Pediatrics, John A. Burns School of Medicine, University of Hawaii, Honolulu, USA; ^2^Department of Pediatrics, John A. Burns School of Medicine, University of Hawai'i, Pediatric Nephrology Kapi‘olani Medical Center for Women and Children, Honolulu, USA; ^3^University of San Francisco, Division of Medical Genetics, San Francisco, USA; ^4^Department of Physiatry, Kapi‘olani Medical Center for Women and Children, Honolulu, USA; ^5^Department of Pediatrics, Kapi‘olani Medical Center for Women and Children, Honolulu, HI, USA

## Abstract

Rhabdomyolysis is diagnosed with creatinine kinase (CK) elevation beyond 1000 U/L or ten times above the normal upper limit. Severe episodes can be fatal from electrolyte imbalance, acute renal failure, and disseminated intravascular coagulation. A 13-month-old child was admitted with a CK of 82,090 U/L in the setting of respiratory tract infection-related hyperthermia of 106.9° farenheit. His medical history was significant for prematurity, dystonia, and recurrent rhabdomyolysis. His home medications clonazepam, clonidine, and baclofen were continued upon admission. He exhibited uncontrolled dystonia despite treatment for dystonia. Therefore, sedative infusions and forced alkaline diuresis were begun to prevent heme pigment-induced renal injury. Despite these interventions, his CK peaked at 145,920 U/L, which is rarely reported in this age group. The patient also developed pulmonary edema despite diuresis and required mechanical ventilation. Sedative infusions were not enough for dystonia management, and he needed the addition of a neuromuscular blocking infusion. He finally responded to these interventions, and the CK normalized after a month. He required a month of mechanical ventilation and two and a half months of hospitalization and extensive rehabilitation. We were able to avert renal replacement therapy despite pulmonary edema and an estimated glomerular filtration rate nadir of 21 mL/min/1.73 m^2^ based on the bedside Schwartz formula. He made a complete recovery and was discharged home. His growth and development were satisfactory for two years after that event. His extensive diagnostic workup was negative. Unfortunately, he died from septic and cardiogenic shock with mild rhabdomyolysis two years later. Prompt recognition, early institution of appropriate therapies, identification of underlying disease, and triggering events are pivotal in rhabdomyolysis management. Evidence-based guidelines are needed in this context.

## 1. Introduction

Rhabdomyolysis is characterized by disruption of the skeletal muscle leading to leakage of intracellular and sarcoplasmic proteins into the circulation. It can present with an asymptomatic elevation of creatinine kinase (CK), mild disease, or severe disease with life-threatening complications such as electrolyte imbalance, arrhythmias, acute renal failure (ARF), and disseminated intravascular coagulation (DIC) [1–3]. The diagnosis of rhabdomyolysis is considered when the CK value exceeds 1000 U/L or at least ten times the normal upper limit [1]. Cardinal causes in the pediatric population include viral myositis, trauma, seizures, physical exertion, medications, intoxication, dystonia, and genetic and metabolic disorders [1–5]. The degree of muscle involvement seems to impact the CK level, and superior levels can be associated with unfavorable outcomes [2, 3, 5]. Acute renal failure has been reported in as high as 42% of cases [3] with up to 11% mortality [4]. The outcomes of rhabdomyolysis can be more detrimental in the pediatric population, although the incidence remains lower than that in adults. Therefore, it is crucial to recognize the underlying etiology and treat it emergently to avoid complications. Extremely high CK levels (as high as 1,000,000 U/L) are typically seen in certain genetic conditions like LPIN-1 mutations [5–8]. Fatal cases have been reported with CK values as low as 22,013 U/L [8]. We present a 13-month-old patient with a CK of 145,920 U/L, a value that is rarely reported in this age group. He made a complete recovery without renal replacement therapy (RRT). We have also discussed the literature review about acute management and diagnostic workup for pediatric patients with severe rhabdomyolysis.

## 2. Case Presentation

A 13-month-old male presented to the emergency department (ED) with fever, vomiting, cough, and trouble breathing for two days. The patient was noted to have hyperpyrexia of 106.9°farenheit, hypoxemia, and dystonia. He received oxygen, an intravenous fluid bolus, an antipyretic, and an antibiotic. Later, he was transferred to the pediatric intensive care unit (PICU) for further management. Vitals in the PICU included ongoing fever of 101˚farenheit, a pulse of 226/min (sinus tachycardia), respiratory rate of 58/min, blood pressure of 106/53 mmHg, and oxygen saturation of 97% with supplemental oxygen. The capillary blood gas showed compensated metabolic acidosis with a pH of 7.317, pCO_2_ of 31.5 mmHg, pO_2_ of 83 mmHg, a base deficit of −9, and bicarbonate of 16.1 mEq/L. His white blood cell count was 24.2 × 10/uL with 52% neutrophils, 23% bands, and 16% leukocytes. A chemistry panel showed sodium of 158 mmol/L, chloride of 122 mmol/L, CO_2_ of 17 mmol/L, blood urea nitrogen (BUN) of 69 mg/dL, creatinine of 1.41 mg/dL, and CK level of 82090 U/L. Chest X-ray showed bilateral infiltrates. His urine analysis showed proteinuria and myoglobinuria.

The patient's past medical history was significant for a month of neonatal intensive care unit (NICU) stay. He was born at 23 weeks of gestational age. He had bronchopulmonary dysplasia (BPD), pulmonary hypertension, intraventricular hemorrhage, global developmental delay, hypotonia, dystonic movements, and gastrostomy tube dependence. After his discharge from the NICU, he had two hospitalizations with viral infections leading to acute rhabdomyolysis with a CK level as high as 5358 U/L. He also exhibited dystonia and spasticity during this time. Therefore, he was started on clonidine, clonazepam, and baclofen sequentially in that order. He responded well, and CK levels were within normal limits afterward. He also remained on sildenafil for his pulmonary hypertension.

In the PICU, his home medications of clonazepam and clonidine were continued, and the dose of baclofen was increased to manage dystonia. Forced alkaline diuresis was started to prevent heme pigment-induced renal injury. This was achieved with one and half times the intravenous maintenance fluids, sodium bicarbonate, mannitol, and other diuretics. Arterial blood gases and urine pH were appropriately maintained. Midazolam and dexmedetomidine infusions were begun to reduce muscle activity and prevent further CK elevation. He exhibited dystonia and spasticity despite sedative infusions, and his CK peaked at 145,920 U/L. With ongoing hydration, the patient also developed pulmonary edema despite diuresis. Given this entire clinical trajectory, he was intubated and paralyzed with vecuronium infusion. Forced alkaline diuresis was continued. During his illness, his BUN peaked at 74, creatinine at 1.41 mg/dL, aspartate aminotransferase at 1886 U/L, alanine aminotransferase 754 U/L, and lactate dehydrogenase 4137 U/L. His estimated glomerular filtration rate (eGFR) nadir was 21 mL/min/1.73 m^2^ by the Schwartz formula using a coefficient of 0.41, and he met the criteria of stage 3 acute kidney injury according to the modified pediatric KDIGO (Kidney Disease Improving Global Outcome) criteria [9].

His PICU course was also significant for infection-related anemia and thrombocytopenia requiring transfusions. He was treated with ceftriaxone for possible secondary bacterial versus viral infection.

After one month, he responded well to all those interventions and eventually showed a decline in CK levels and resolution of the end-organ dysfunction. He also required a month of mechanical ventilation and two and a half months of hospitalization with extensive rehabilitation.

The workup for rhabdomyolysis included urine organic acid, plasma amino acid, carnitine/acylcarnitine profile, biotinidase, thiamine, copper, ceruloplasmin levels, fatty acid levels, and purine/pyrimidine panels, which were all within normal limits. Urine organic acid and plasma amino acid were obtained twice during acute rhabdomyolysis episode. Serum ammonia was obtained on several occasions during acute rhabdomyolysis phase. The other metabolic workup was obtained only once during the acute episode of rhabdomyolysis. Since they were obtained during the peak of the illness, they were thought to be informative and therefore were not repeated. His EEG was normal. A magnetic resonance imaging of his brain done 2 weeks prior to this admission showed chronic sequelae of intraventricular hemorrhage without any acute intracranial findings. The other rare conditions causing hyperkinetic movement disorders like CSF neurotransmitter disorders, channelopathies, disorders of GABA metabolism, and other mutations were ruled out by CSF studies. The workup included normal CSF concentrations of 3-O-methyldopa and 5-methyltetrahydrofolate (MTHF) levels and normal levels of homovanillic acid and 5-hydroxyindoleacetic acid levels. His rhabdomyolysis genetic panel, including multiple genes for glycogen storage disorders and fatty acid oxidation defects, was normal. Chromosomal microarray suggested an increased risk for recessive disorders due to runs of homozygosity.

Whole-exome sequencing showed variants of unknown significance in the following genes: OTC (associated with ornithine transcarbamylase deficiency, an X-linked disorder) hemizygous c.374C>T; DMD (associated with Duchenne/Becker muscular dystrophy, an X-linked spectrum of disorders) hemizygous c.1682G>C; SMARCA2 (SWI/SNF related, matrix-associated, actin-dependent regulator of chromatin, subfamily a, member 2 associated with Nicolaides–Baraitser syndrome, an autosomal dominant condition) heterozygous c.3344C>T; COL12A1 (collagen type XII, Alpha1 associated with Bethlem-like myopathy and congenital myopathies, recessive and dominant disorders) heterozygous c.6060G>A; and NEFH (neuro filament protein heavy polypeptide associated with Charcot–Marie–Tooth disease type 2CC, a dominant disorder) heterozygous c.1138G>A.

The patient regained his baseline status, and he was discharged home with a plan to follow up with cardiology, neurology, and genetics with the possible performance of a muscle biopsy and family genetic evaluation.

### 2.1. Follow-Up

One year later, the patient was again hospitalized with a parainfluenza infection. He did not exhibit rhabdomyolysis at this time despite acute illness. Another three months after, he again demonstrated a normal CK and a baseline neurological exam upon a follow-up appointment with the neurologist. He gained approximately three kilograms weight in two years, and his development was on a satisfactory trajectory for his condition. His spasticity had resolved, and weaning plans were made for clonazepam and baclofen. His follow-up echocardiogram showed ongoing mild pulmonary hypertension without any other abnormalities.

Two years after this initial severe rhabdomyolysis episode, he was again admitted to the PICU in a critically ill condition with shock and lactic acidosis. He was found to have a respiratory syncytial virus infection. His CK at that time was 2989 U/L. His echo revealed severely decreased left ventricular (LV) systolic function with global hypokinesis, with an estimated LV ejection fraction of around 20%. The left ventricle was slightly enlarged. His VBG showed a pH of 7.13, pCO_2_ of 58, and HCO_3_ of 19, with BE of −10.9, lactate of 7.1, BUN of 33, creatinine of 1.22, and a 10% bandemia. He died from septic and cardiogenic shock with pulseless electrical activity. The autopsy revealed chronic pneumonitis with necrosis and congestion, BPD, diffuse interalveolar hemorrhage, old and new hemorrhagic brain infarcts including watershed injury, biventricular cardiac hypertrophy with ecchymosis and petechiae, congested kidneys, and edema and erythema of the right calf with otherwise normal histology. An overall histopathology was consistent with necrotizing pneumonitis, shock, and end-organ dysfunction.

## 3. Discussion

This manuscript presents a case of severe rhabdomyolysis in a thirteen-month-old patient. This degree of rhabdomyolysis with a CK value of 145,920 U/L has been rarely reported in this age group, and not all of them made a complete recovery without renal replacement therapy [2–4]. Forced alkaline diuresis was helpful in our patient, but it also led to pulmonary edema. His CK continued to rise, and his lowest eGFR was 21 mL/min/1.73 m^2^. This put him in stage 3 acute kidney injury based on the Kidney Disease: Improving Global Outcomes (KDIGO) guidelines [9]. We used sedation and paralysis to control his dystonia and spasticity, with which we were able to avert CRRT despite this severe condition. The mechanism of sedation and paralysis for treating dystonia and rhabdomyolysis is intelligible but has been rarely reported in the literature.

Forced diuresis to reduce the risk of acute renal injury is the cornerstone of immediate treatment of rhabdomyolysis. Mannitol is a commonly used diuretic because of its ability to increase renal blood flow and glomerular filtration rate. Mannitol's osmotic properties may reduce muscular swelling and nerve compression by drawing fluid out of the interstitial compartment and increasing urine flow to prevent myoglobin cast formation. Lastly, mannitol has been suggested to scavenge free radicals [1, 10, 11]. Other diuretics are indicated in the presence of fluid overload, like in our patient, or to turn anuric renal failure into oliguric [10]. Renal replacement therapy or hemodialysis may not directly impact acute rhabdomyolysis because of ineffective myoglobin removal, although it may reduce the burden of myoglobin. However, it may be beneficial in patients with life-threatening electrolyte abnormalities, acidosis, and fluid overload [1, 2, 4, 10]. In one study, the peak CK value of more than 10,000 U/L was associated with a high likelihood of RRT requirement [2].

Identifying underlying conditions leading to severe and recurrent cases is also essential. Typical etiologies responsible for acute rhabdomyolysis are infection, trauma, intoxication, medication adversity, and exercise. A careful effort must be carried out to identify these common causes. Lack of these common etiologies should lead to workup geared towards rare causes, including genetic, metabolic, immune, endocrine, and neurologic disorders. Although there are no evidence-based consensus guidelines on diagnostic workup, various authors have suggested some pathways [1, 4, 10]. Since our patient's initial workup was negative, a whole genetic exome was ordered, which showed five gene variants, including DMD, OTC, SMARCA2, COL12A1, and NEFH. These were multiple variants of unknown significance. Out of those 5, only variants in the DMD gene are known to be associated with rhabdomyolysis. The other four variants found in our patient have not been reported in such a context [1, 4, 10].

DMD genes are typically associated with X-linked cardiomyopathy and Becker and Duchenne muscular dystrophy. The specific variant (c.1682G>C, also written as p.Trp561Ser) identified in the DMD gene in our patient was novel and has not been reported previously in the HGMD or Broad ExAc data set of >6,000 individuals without severe childhood-onset disease. The variant was a substitution of the amino acid serine for tryptophan. The physiochemical differences between serine and tryptophan are considered significant, with a Grantham distance of 177 (0–215). Computational tools for the effect of missense alteration predict: Align GVGD = likely benign and SIFT = tolerated. The amino acid substitution of serine for tryptophan at position 561 is seen in 0/10 primates, 0/60 mammals, and 0/95 evolutionarily more distant species. Following the American College of Medical Genetics and Genomics guidelines, this variant was classified as a variant of uncertain significance as the above data are not conclusive as a disease-causing variant [12, 13]. Our patient's CK level varied between being significantly elevated and normal, which is less commonly reported in DMD. CK levels are typically elevated at birth (day 1), remain elevated at day 4 of birth [14], and after that throughout life. CK levels in DMD might decrease later in life in the late teenage years to above normal levels as muscle mass decreases as it is replaced throughout the years by fibrosis and adipose tissue as part of the natural history of DMD. The significant variability in CK levels in our patient, its association with acute concurrent illnesses, and his presentation starting in the neonatal age might correspond to an underlying metabolic disorder capable of causing an intermittent metabolic crisis with significant rhabdomyolysis, which is exacerbated further with his additional underlying diagnosis of cerebral palsy, prematurity, and intraventricular hemorrhage with hypertonia (spasticity and dystonia).

Our patient's autopsy revealed cardiac biventricular hypertrophy, but otherwise normal skeletal muscle histology. Immunohistochemical analysis for the dystrophin gene was not performed. However, if his disease were severe enough to cause this degree of rhabdomyolysis, cardiac hypertrophy, and death, muscular histology would have shown some changes consistent with DMD.

Given his negative extensive workup, it is possible that he had dystonia-related rhabdomyolysis, which has been reported before [1, 3, 4, 10]. The degree of CK elevation in our patient triggered the extensive workup, which ruled out other conditions. Therefore, his diagnosis of dystonia-related rhabdomyolysis was clinical and a diagnosis of exclusion. This degree of rhabdomyolysis from dystonia in this age group has been rarely reported in the literature to the best of our knowledge. His complete recovery makes it more noteworthy. His death later was probably unrelated to rhabdomyolysis. However, the recurrent nature of rhabdomyolysis in our patient typically triggered by acute infection, along with lactic acidosis and cardiac hypertrophy noted on autopsy, raises concerns for a possible undiagnosed genetic condition. This case report also illustrates an extensive battery of investigations needed in such cases in a stepwise fashion.

We also did an extensive literature review to identify pediatric cases reporting rhabdomyolysis. A PubMed search was conducted for the terms “infant rhabdomyolysis,” “infant CPK,” “infant CK,” “rhabdomyolysis pediatrics,” and “rhabdomyolysis children.”

This search found a total of 24 case reports of severe rhabdomyolysis in infants and 21 case reports in children with 1-2 years of age. We also found two case series about pediatric rhabdomyolysis. We reviewed cases in depth if they reported mortality (15 cases), dialysis requirement (12 cases), and cases reporting a CK value higher than ours (5 cases).

One death was reported from drowning in a two-year-old, and rhabdomyolysis was associated with the CK of 76,826 U/L [15]. Two deaths were associated with anesthesia-induced malignant hyperthermia and rhabdomyolysis. CK in these reports was 16,400 in a one-year-old and 17,700 U/L in a four-month-old [16, 17]. The fourth death was reported in an 18-month-old patient, which was related to acute renal failure due to tacrolimus-associated rhabdomyolysis. Although the patient also had grade III acute GVHD, BUN was 20, creatinine was 0.6, and CK was 550 IU/L, it remains unclear if the death was solely attributable to rhabdomyolysis [18]. Overall, these cases had lower CK values than our patient. The etiology of rhabdomyolysis was associated with mortality.

Five deaths were reported in patients with LPIN-1 mutation. Only one infant in this series whose CK was over 20,000 U/L died. One child younger than two years of age had a CK of 900,000 U/L but survived. The highest reported CK was 10,000,000 U/L in an eight-year-old in this series with LPIN-1 mutation. Four other patients in this study had CK levels higher than ours, but they all were older than three years of age. The rest of the patients had a CK level lower than ours. Fever, fasting, and local anesthetics were common triggering events. The manuscript did not describe the treatment options but recommended genetic and metabolic workup for rhabdomyolysis followed by a muscle biopsy [5].

Six other deaths were reported in a study of 55 pediatric patients with rhabdomyolysis. There were seven infants in this study. The study reported a variety of genetic and nongenetic conditions leading to rhabdomyolysis. The highest CK level was 126,994 IU/L in a two-year-old with TANGO2-related disorder, which was lower than ours. There were seven infants in this study, one of them died, and the rest six required forced alkaline diuresis or RRT. A total of 11 other pediatric cases required RRT, and none of them had a CK level higher than our patient [4].

A dialysis requirement was also reported in an eleven-month-old patient with pontocerebellar hypoplasia type 2 with a CK level as high as 258,200 U/L [19].

A 2-year-old patient had a CK of 370,448 U/L after anaphylaxis and rhabdomyolysis after fire ant envenomation. His BUN peaked at 49, and creatinine at 2.6. He responded to fluid resuscitation, dopamine, and sodium bicarbonate infusion. They were also able to avert dialysis, similar to our case. However, again, this patient was older than ours [20].

A recent case report of a 16-year-old male with a CK of 427,656 U/L associated with COVID-19 infection [21]. He exhibited normal renal function and was discharged within 12 days. Since the CK level is related to the muscle mass involvement, presumably, the younger infant would have a lower muscle mass than the older children. Therefore, a CK level of 145,920 U/L in our patient with renal failure and pulmonary edema was indicative of severe disease.

Overall, our literature search for these other cases revealed that unfavorable outcomes were associated with one of the three factors, including superior CK values, underlying conditions, and inciting events.

The following lessons were learned through this case, and we hope to spread this message to improve outcomes in such cases.

## 4. Conclusions

Acute rhabdomyolysis can be lethal. Prompt recognition and early institution of appropriate therapies are critical. Forced diuresis should be considered early on. Consideration should be given to mannitol use and alkalinization, although the data remain insufficient in this context. Sedation and paralysis were helpful in the management of severe dystonia-related rhabdomyolysis in our patient. More extensive studies are needed to explore the yield. Identifying the underlying disease and triggering events are the next imperative steps in managing acute rhabdomyolysis. Appropriate workup should be carried out as discussed in our case. Evidence-based guidelines are needed in this field.

## Data Availability

The CASE REPORT data used to support the findings of this study are included within the article.
